# Cognitive Training Effectiveness on Memory, Executive Functioning, and Processing Speed in Individuals With Substance Use Disorders: A Systematic Review

**DOI:** 10.3389/fpsyg.2021.730165

**Published:** 2021-08-13

**Authors:** Tânia Caetano, Maria Salomé Pinho, Eduardo Ramadas, Cátia Clara, Timóteo Areosa, Maria dos Anjos Dixe

**Affiliations:** ^1^Center for Research in Neuropsychology and Cognitive and Behavioral Intervention, Faculty of Psychology and Educational Sciences, University of Coimbra, Coimbra, Portugal; ^2^Center for Innovative Care and Health Technology (ciTechcare), Polytechnic of Leiria, Leiria, Portugal; ^3^VillaRamadas International Treatment Centre, Research and Innovation Department, Leiria, Portugal

**Keywords:** cognitive training, executive functioning, memory, processing speed, substance use disorders, systematic review

## Abstract

**Background:** Cerebral neuroplasticity is compromised due to substance abuse. There is damage to neuronal areas that are involved in memory and executive functioning. Treatments with worse outcomes are often associated with cognitive deficits that have resulted from substance dependence. However, there is evidence that cognitive training can lead to improvements in cognitive functions and can be useful when treating addictions. This systematic review aims to synthesize evidence on the effectiveness of cognitive training in memory, executive functioning, and processing speed in individuals with substance use disorder (SUD).

**Methods:** The Joanna Briggs Institute's PICO strategy was used to develop this systematic literature review. Four databases were searched (PubMed, the Cochrane Library, Web of Science, and PsycINFO) to identify controlled randomized clinical studies and quasi-experimental studies, in English, Portuguese, and Spanish, from 1985 to 2019. The literature found was examined by two independent reviewers, who assessed the quality of studies that met the inclusion criteria. The Cochrane risk-of-bias tool for the randomized controlled trials and the ROBINS-I tool for non-randomized studies were used to assess the risk of bias. In data extraction, the Cochrane Handbook for Systematic Reviews was considered.

**Results:** From a total of 470 studies, 319 were selected for analysis after the elimination of duplicates. According to the inclusion criteria defined, 26 studies were eligible and evaluated. An evaluation was performed considering the participant characteristics, countries, substance type, study and intervention details, and key findings. Of the 26 selected studies, 14 considered only alcoholics, six included participants with various SUD (alcohol and other substances), three exclusively looked into methamphetamine-consuming users and another three into opioid/methadone users. Moreover, 18 studies found some kind of cognitive improvement, with two of these reporting only marginally significant effects. One study found improvements only in measures similar to the training tasks, and two others had ambiguous results.

**Conclusions:** The included studies revealed the benefits of cognitive training with regard to improving cognitive functions in individuals with SUD. Memory was the most scrutinized cognitive function in this type of intervention, and it is also one of the areas most affected by substance use.

**Systematic Review Registration:** [PROSPERO], identifier [CRD42020161039].

## Highlights

- Substance dependence is associated with impaired memory, executive functioning, and processing speed.
- Cognitive training can contribute to improvements in cognitive functions in individuals with substance use disorders.
- Addiction treatment can benefit from cognitive training since it can improve cognitive functions and addiction symptoms and decreases the likelihood of relapse.

## Introduction

Substance abuse is a worldwide problem. It has not only medical, but also social and economic consequences. According to the World Health Organization (2018), it is estimated that 31 million people experience substance use disorders (SUD) and that annually 3.3 million die due to harmful use of alcohol alone. Despite this, presently adequate treatment is only accessible to a minority (Ozgen and Blume, 2019).

Addiction is characterized by a disruption in the brain's reward system cycle, which tends to increase progressively and lead to compulsive consumption of a certain substance, therewith leading to loss of control (Koob and Moal, 1997). Progress in neuroscience has allowed the conceptualization of addiction as a chronic brain disease that comprises several factors, among which are socio-cultural, genetic, and even neurodevelopmental features (Volkow and Morales, 2015). Substance dependence or repeated drug use compromises the neuroplasticity of the brain. Several regions of the brain are impaired due to this consumption, including the neural areas involved in memory (Fernández-Serrano et al., 2011; Sampedro-Piquero et al., 2019) and executive functioning (Fernández-Serrano et al., 2011; Morie et al., 2014). Continued substance use impairs brain function, interfering with self-control and making the subject more sensitive to high stress levels and more prone to the presence of negative mood (Volkow and Morales, 2015). Addiction is also characterized by compulsive behaviors (Volkow and Morales, 2015).

When an individual becomes addicted to a particular substance, nerve cells that are located in the brain's reward circuit tend to adapt epigenetically during repeated exposure to the substance in question. These adaptations lead to lasting changes in brain functions, which in turn contribute to dysfunctional behaviors related to the abused substance (Hamilton and Nestler, 2019). In fact, cognitive impairment resulting from substance use is not only common but has been linked to worse treatment outcomes (Sampedro-Piquero et al., 2019).

According to several authors [Vonmoos et al., 2014; see Sampedro-Piquero et al. (2019)], cognitive impairment that results from substance use can be reversed, at least partially, by prolonged drug withdrawal. Abstinence reinforces the neuroplasticity of the brain and, therefore, its regenerative capacity (Sampedro-Piquero et al., 2019). However, others (e.g., Volkow and Morales, 2015; Verdejo-Garcia, 2016) propose that interventions that improve cognitive functioning can contribute to the long-term success of treatment for addiction. Volkow and Morales (2015) go so far as to say that these interventions would be useful even if total abstinence does not occur.

As Hofmann et al. (2012) described, impairment in core executive functions has been linked to poor self-regulation and decision-making. Working memory (WM) impairments, for example, could not only interfere with patient's daily activities (e.g., finding and holding a job) but also impact important clinical variables, such as dropout rates (Rezapour et al., 2016). Such impairments can also make it harder for individuals to correctly evaluate high-risk situations, which may then result in greater difficulties preventing relapse or achieving personal goals (Rochat and Khazaal, 2019). As such, it is not surprising that neurocognitive impairments have been growingly considered as relevant transdiagnostic targets for SUD treatment (Yücel et al., 2019). Interventions that aim to reduce cognitive impairment in these domains, namely cognitive training, could lead to improved treatment outcomes.

There are many types of cognitive training programs, such as working memory training (WMT), executive-functions training, video-game training, and even music and chess instruction (Sala and Gobet, 2019). Working memory training is the most studied type of cognitive training programs (Sala and Gobet, 2019), and its predominance can be explained by the known association between WM and fluid and general intelligence (Salthouse and Pink, 2008). Given its essential role in cognition, it has been believed that WMT could lead to improvements in domain-general cognitive skills and, as such, allow for “far-transfer” of training effects. These programs tend to be structured (e.g., number and duration of sessions) and make use of specialized computer software, but they can differ with regards to the specific structure, the chosen tasks (e.g., *n*-back tasks) and the difficulty level. Executive-functions training programs, similarly to WMT, tend to be structured but propose to focus on more than one cognitive domain. Beyond WM, these programs can also consider training tasks concerning inhibitory control and cognitive flexibility, as well as, reasoning and problem-solving skills (Diamond, 2013). While in WMT, most programs are computerized, in executive-functions training there seems to be a higher heterogeneity with regards to the context and delivery of the chosen tasks (e.g., computer-based tasks, add-ons to school curriculum, martial arts programs; Diamond, 2013). While WMT and executive-functions training tend to be programs specifically designed with the goal of improving cognitive functioning, it was hypothesized that other, less specific but cognitively demanding activities could have similar benefits. Among them, videogames, music and chess instruction have all received considerable scientific interest and been the subject of several studies (Sala and Gobet, 2019). Despite the diversity of cognitive training programs, overall cognitive training is thought to produce both functional and anatomical changes in the neural system that lead to improvement in cognitive function (Sala and Gobet, 2019).

Although the potential value of improving cognitive functioning in certain populations such as SUD is not disputed, there is disagreement concerning the use of cognitive training for this end. There is an on-going controversy surrounding the effectiveness and clinical relevance of cognitive training that lies on the question: Is it possible for domain-specific tasks and training to impact domain-general cognitive skills? Many studies have cast doubt to the possibility of “far transfer” of any effects resulting from cognitive training (e.g., Melby-Lervåg and Hulme, 2013; Melby-Lervåg et al., 2016; Redick, 2019; Sala and Gobet, 2019), indicating that these effects tend to be short-term and/or training specific, and therefore don't lead to generalized cognitive benefits. Sala and Gobet (2019) go further and argue that when significant effects are observed, they are often associated with limitations in the design of the experiments, such as the lack of an active control group. However, there is the argument that the longevity or “far-transfer” effects of cognitive training could be being masked by the studies' almost exclusive reliance on primary outcomes, as suggested by Brooks et al. (2020) in regards to WMT. These authors also postulate that the current definition of “far-transfer” is too narrow, since it does not consider how cognitive performance (e.g., WM performance) might impact apparently unrelated functions (e.g., impulse control). In fact, in a review of the neural processes of WMT, Brooks et al. (2020), reported that significant neural effects (in frontoparietal and frontostriatal circuitry) could be found, often independently of behavioral changes. Moreover, they reported that alongside neural changes, various neuroimaging studies found “far-transfer” effects of WMT to other un-related cognitive domains.

The on-going debate highlights the importance that more studies be conducted with the aim of reviewing the effectiveness of cognitive training programs on specific contexts and populations, such as SUD.

In the present systematic review, we aim to understand whether cognitive training interventions are effective in improving memory and/or executive functioning in individuals with SUD. In this sense, we intended to synthesize the effectiveness of cognitive training in individuals with SUD with regard to improving memory, executive functioning, and processing speed by answering the following questions:

I Is it possible to improve the memory of individuals with SUD through cognitive training programs?
II Is it possible to improve the executive functioning of individuals with SUD through cognitive training programs?
III What are the most used cognitive training programs in individuals with SUD and what is their effectiveness?

## Methods

### Search Strategy

The protocol for this review was registered and published in the International Prospective Register of Systematic Reviews (PROSPERO) with identification number CRD42020161039.

The Population, Intervention, Comparison, and Outcome (PICO) strategy of the Joanna Briggs Institute (JBI; Aromataris and Munn, 2017) was the basis for this systematic literature review. The main objective was to synthesize the effectiveness of cognitive training in individuals with SUD when there are improvements in memory, executive functioning, and processing speed. The research strategy aimed to identify published studies, as well as unpublished studies, written in English, Portuguese, and Spanish, from 1985 to 2019. The selected period was based on the first found article referring to cognitive training in individuals with SUD (Godfrey et al., 1985). It was also intended to include gray literature to limit the bias of the present review.

Initially, a general search was carried out in the JBI Database of Systematic Reviews and Implementation Reports, the Cochrane Database of Systematic Reviews, MEDLINE, Epistemonikos, and PROSPERO to confirm the absence of other systematic literature reviews with the same objectives as the present review. Subsequently, an exhaustive and limited search in four databases was performed, including PubMed, the Cochrane Library, Web of Science, and PsycINFO. Then, the titles were analyzed and the articles were summarized using the search terms.

The search terms originated from DeCS® and Medical Subject Headings (MeSH Browser®). These were also combined with the Boolean operators, as well as with the elements of the PICO strategy. Below are the keywords used in the search: *Substance-Related Disorders, Addiction Medicine, Alcoholism, Alcohol Abuse, Alcohol Dependence, Substance Abuse, Addiction Treatment, Drug Abusers, Drug Abuse, Cocaine Abusers, Cocaine Dependent, Cocaine-Related Disorders, Marijuana Abuse, Opioid-Related Disorders, Amphetamine-Related Disorders, Hallucinogens*, Subst*ance-Use Disorders, Problem Substance Use, Drug Dependence, Cognitive Stimulation Program, Cognitive Stimulation, Cognitive Rehabilitation, Cognitive Training, Memory Training, Cognitive Intervention, Brain Training, Executive Training, Neurocognitive Training, Reasoning Training, Mental Training*.

The Boolean operators were arranged as follows:

(*Substance-Related Disorders* OR *Addiction Medicine* OR *Alcoholism* OR *Alcohol Abuse* OR *Alcohol Dependence* OR *Substance Abuse* OR *Addiction Treatment* OR *Drug Abusers* OR *Drug Abuse* OR *Cocaine Abusers* OR *Cocaine Dependent* OR *Cocaine-Related Disorders* OR *Marijuana Abuse* OR *Opioid-Related Disorders* OR *Amphetamine-Related Disorders* OR *Hallucinogens* OR Subst*ance-Use Disorders* OR *Problem Substance Use* OR *Drug Dependence*) AND (*Cognitive Stimulation Program* OR *Cognitive Stimulation* OR *Cognitive Rehabilitation* OR *Cognitive Training* OR *Memory Training* OR *Cognitive Intervention* OR *Brain Training* OR *Executive Training* OR *Neurocognitive Training* OR *Reasoning Training* OR *Mental Training*). This survey was conducted between May and 31st of July 2020.

Lastly, the references of all selected studies were analyzed for the possibility of including new studies. The articles resulting from the bibliographic search, organized according to the steps previously described, were analyzed by two reviewers. First, the titles and abstracts of studies that could possibly be eligible for the literature review were evaluated, followed by the analysis of the full article.

### Selection Criteria

#### Inclusion Criteria

##### Types of Participants

The present review aimed to select studies that included individuals with SUD, aged ≥ 18 years.

##### Types of Intervention(s)

In this review were included studies on cognitive training programs focused on memory and/or executive functioning in individuals with SUD. Moreover, since the terms cognitive training, stimulation, and rehabilitation are often confused and used interchangeably in the literature, studies on programs with these designations (i.e., stimulation or rehabilitation) were also considered, provided their characteristics were in line with the description of cognitive training presented below. Cognitive training, which is the focus of the present review, usually entails guided practice on a number of structured tasks that focus on specific cognitive functions (e.g., memory, attention), and can be applied individually or in a group. It is common for tasks to present different levels of difficulty, allowing the selection of the appropriate level for each individual. This type of intervention is grounded on the assumption that regular practice tends to improve or, if improvement is not possible, maintain functioning in a certain cognitive domain, and possibly allow the generalization of cognitive gains over time. As a rule, the results are assessed using cognitive or neuropsychological instruments (Clare and Woods, 2004). Contrastingly, cognitive stimulation generally involves a series of tasks/activities and discussions in a group context, with the intention of improving not only cognitive but also social functioning. This type of approach concerns a generalist method, with no focus on specific cognitive functions, since it is based on the argument that cognitive functions should not be exercised in isolation, but rather combined with other functions (Clare and Woods, 2004). Finally, in cognitive rehabilitation, there is an individualized approach in which the individual, and sometimes their family, helps to establish personally-relevant goals and device appropriate strategies for their particular experience and social context. The focus is on improving the functioning on the everyday context and not on specific cognitive tasks. In this case, neuropsychological tests are not used with the aim of observing improvements in cognitive functions, but rather to substantiate any impact that may result from the changes inherent to the disease in question (Clare and Woods, 2004).

##### Types of Results

This review aimed to include studies that considered cognitive training programs, namely for (working and long-term) memory, executive functioning (planning, abstract reasoning, cognitive flexibility, and inhibitory control), and processing speed.

##### Types of Studies

The selected studies were experimental (randomized controlled, and quasi-experimental with a control group) in an adult population, with articles written in English, Spanish, or Portuguese. The studies had to meet the following inclusion criteria:

a) a control group that has the same characteristics as the experimental group (individuals with SUD, aged ≥ 18 years);
b) pre- and post-test evaluations;
c) objective measures to assess memory and/or executive functioning; and
d) standardized measures (in the pre- and post-tests) that are not the same or identical to the exercises used in the cognitive training.

##### Controls

This review included studies with an active or a passive control group. An active control group is identified by the consideration that another type of intervention is performed on the participants, without affecting the variables of interest, such as the same intervention with some changes (alternative intervention) or another type of intervention. In the inactive/passive control group, participants are not subjected to any other type of intervention and/or treatment or alternatively are subjected to standard care (e.g., treatment as usual) or a placebo (Karlsson and Bergmark, 2015; Coughtrey et al., 2018).

#### Exclusion Criteria

All studies that were not published in English, Spanish, or Portuguese were excluded. Review studies and animal studies were also excluded.

### Evaluation of the Methodological Quality of the Studies

The identified articles were independently evaluated by two reviewers, using the standardized JBI instruments. In this context, we used the JBI Critical Appraisal Checklist for Randomized Controlled Trials for randomized controlled trials and the JBI Critical Appraisal Checklist for Quasi-Experimental Studies (non-randomized experimental studies) for quasi-experimental studies (Tufanaru et al., 2017).

To assess the quality of a study, namely the risk of bias, we used the Cochrane risk-of-bias tool for the randomized controlled trials (Higgins et al., 2011). This checklist allowed us to perform a complete assessment of risk of bias that may affect the cumulative evidence of the review. Six bias domains were examined: selection, performance, detection, attrition, reporting, and other biases. The studies were classified as “unclear risk,” “low risk,” and “high risk” in each of the above domains. In turn, for the non-randomized studies, the Risk of Bias in Non-randomized Studies - of Interventions (ROBINS-I) tool was used (Sterne et al., 2016). The following domains were analyzed: baseline confounding, selection of participants, classification of intervention, deviation from intended intervention, missing data, measurement of outcomes, and selection of reported results. In this case, each study in question was classified as “low risk of bias,” “moderate risk of bias,” “serious risk of bias,” “critical risk of bias,” and “no information.”

In situations where the reviewers did not reach a consensus on the inclusion or exclusion of a study, a third reviewer intervened. All studies that met the inclusion criteria are included in this review, and any methodological weaknesses present in the selected studies are also discussed.

### Data Extraction

Data were extracted considering the *Cochrane Handbook for Systematic Reviews* (Li et al., 2020). Analysis considered the following items:

1. Countries
2. Substance type
3. Randomization and blindness
4. Cognitive functions
5. Follow-up
6. Outcome measures
7. Characteristics of interventions
8. Key findings

The data were extracted by two independent reviewers (TC; ER).

### Data Synthesis

Due to the heterogeneity of the data, no meta-analysis was performed. Therefore, a narrative approach was used for data synthesis. There were significant differences between interventions, populations, comparators, and the presentation of outcome results, and thus it was not possible to make a direct comparison regarding the study results. Since statistical pooling was not viable, it was then decided to use tabular and narrative formats to present the results.

## Results

### Study Selection and Search Results

**Figure 1:**
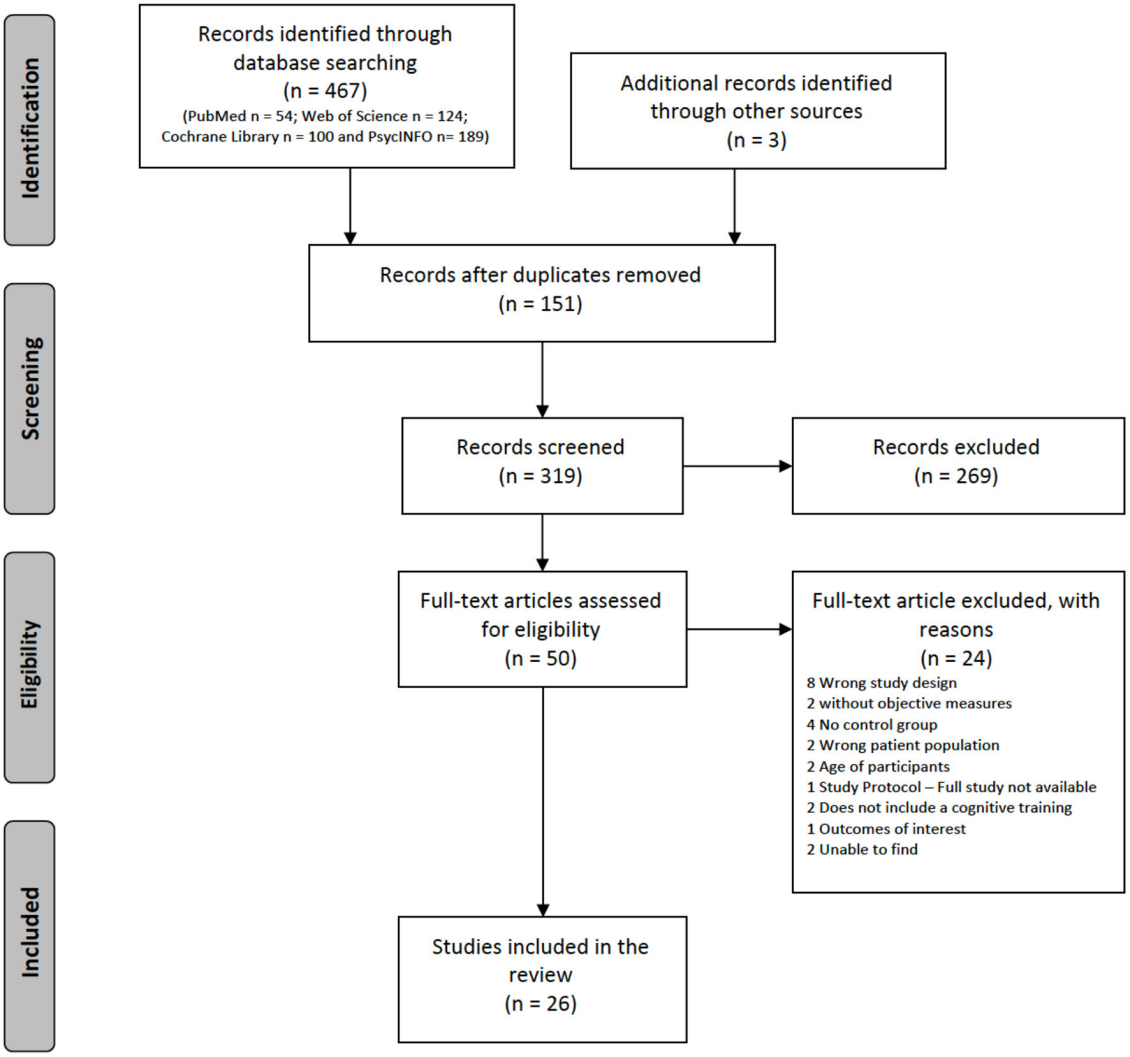
Preferred reporting items for systematic reviews and meta-analyses (PRISMA) flow diagram.

The Preferred Reporting Items for Systematic Reviews and Meta-Analyses (PRISMA) flowchart (see Figure 1) shows the studies included and excluded from the present review. Through the research strategies identified above, a total of 467 studies were obtained (54 in PubMed, 124 in Web of Science, 100 in the Cochrane Library, and 189 in PsycINFO) and three studies using other research sources. After the elimination of duplicates, 319 studies remained for analysis. To determine the eligibility of the studies according to the inclusion criteria, their titles and abstracts were analyzed. Fifty studies were considered based on the eligibility criteria; they were analyzed in full by two reviewers (TC; ER). In case of discrepancies, a third reviewer intervened (TA). After this analysis, 24 studies were excluded (see Supplementary Material) and 26 studies met all inclusion criteria. Of the 26 studies included, 25 are controlled randomized clinical studies (Godfrey and Knight, 1985; Godfrey et al., 1985; Yohman et al., 1988; Wetzig and Hardin, 1990; Fals-Stewart and Lucente, 1994; Steingass et al., 1994; Peterson et al., 2002; Goldstein et al., 2005; Fals-Stewart and Lam, 2010; Rupp et al., 2012; Gamito et al., 2013, 2014, 2016, 2017; Eack et al., 2015; Rass et al., 2015; Bell et al., 2016, 2017; Hendershot et al., 2018; Zhu et al., 2018; Khemiri et al., 2019; Rezapour et al., 2019) and one is quasi-experimental (Hannon et al., 1989). The PRISMA guidelines were used to conduct this systematic literature review.

### Participant Characteristics

Table 1 summarizes the characteristics of the participants from the 26 included studies. The sample sizes ranged from 12 to 160 participants (with an average of 56.5 participants).

**Table 1:** Subject characteristics.

**References**	**Country**	**Population**	***n***	**Age (M ± SD), years**	**Gender** **(% male)**	**Drug**
Godfrey et al. (1985)	New Zeland	Amnesic alcoholic patients	17	52.3 in the experimental group 60.2 in the activation control group 61.3 in the no-treatment group	± 71	Alcohol
Godfrey and Knight (1985)	New Zeland	Amnesic alcoholic patients	12	57.1 ± 12.5	NP	Alcohol
Yohman et al. (1988)	United States	Alcoholic subjects	76	42.5 ± 9.7 in the experimental group 43.0 ± 8.5 in the control group	100	Alcohol
Hannon et al. (1989)	United States	Alcoholics	29	42.6 ± 11.6 in the experimental group 43.3 ± 9.7 in the control group	100	Alcohol
Wetzig and Hardin (1990)	United States	Patients with AUD	45	34.7 ± 9.8 in the experimental group 37.0 ± 12.3 in the practice group 32.6 ± 10.5 in the control group	100	Alcohol
Steingass et al. (1994)	Germany	Alcoholics	29	52.72 ± 8.29 in the experimental group 52.24 ± 7.91 in the control group	± 83	Alcohol
Fals-Stewart and Lucente (1994)	United States	Patients with SUD	72	29.3 ± 6.0	74	Alcohol, cocaine, opioids, stimulants, cannabis
Peterson et al. (2002)	United States	Inpatients detoxified from AUD	38	45.0 ± 4.04 in the cog-rem group 4± 7.43 in the tape group 47.25 ± 7.34 in the control group	NP	Alcohol
Goldstein et al. (2005)	United States	Inpatients with alcoholism	40	NP	NP	Alcohol
Fals-Stewart and Lam (2010)	United States	Patients with SUD	160	32.4 ± 7.1 in the experimental group 33.1 ± 6.8 in the control group	± 59	Alcohol, cannabis, cocaine, opiates, stimulants, other
Rupp et al. (2012)	Austria	Patients with alcohol dependence	41	45.2 ± 10.5 in the experimental group 45.5 ± 8.8 in the control group	± 63	Alcohol
Gamito et al. (2013)	Portugal	Patients with ADS	30	45.73 ± 10.77	± 83	Alcohol
Gamito et al. (2014)	Portugal	Patients ADS	68	45.5 ± 10.18 in the experimental group 45.25 ±10.26 in the control group	± 66	Alcohol
Eack et al. (2015)	United States	Patients misusing substances with schizophrenia or with schizoaffective disorder	31	34.67 ± 12.99 in the control group 39.68 ± 13.64 in the experimental group	± 71	Alcohol/Cannabis
Rass et al. (2015)	United States	Methadone maintenance patients	56	43.3 ± 8.8 in the experimental group 43.5 ± 7.1 in the control group	± 46	Methadone
Bell et al. (2016)	United States	Older veterans with AUD	31	55.27 ± 5.27 in cognitive training + work therapy 55.06 ± 5.23 in work therapy only	96,7	Alcohol, Opioids or Cocaine
Brooks et al. (2016)	South Africa	MA patients	66	28.00 ± 6.132 in baseline CT 29.00 ± 6.291 in baseline TAU 27.67 ± 8.714 in healthy control	100	Methamphetamine
Gamito et al. (2016)	Portugal	Patients with alcohol dependence syndrome	42	45.45 ± 10.3	± 90	Alcohol
Bell et al. (2017)	United States	Older veterans with SUD	48	51.3 ± 9.7 in cognitive training + work therapy 53.8 ± 7.4 in work therapy only	± 94	Alcohol, Opioids or Cocaine
Brooks et al. (2017)	South Africa	MUD in-patients	60	(28.11 ± 6.01) in TAU group (29.83 ± 7.32) in CT group (27.67 ± 8.714) in control group	NP	Methamphetamine
Gamito et al. (2017)	Portugal	Heroin users diagnosed with dependence use disorder	14	37 ± 4.48	100	Heroin, Methadone
Gunn et al. (2018)	United States	Individuals with AUD	145	21.80 ± 2.14 in AUD group 22.30 ± 2.64 in the control group	40	Alcohol
Hendershot et al. (2018)	Canada	Inpatients with SUD	110	39.40 ± 11.42 in the adaptive group 40.00 ± 11.19 in the non-adaptive group	± 69	Alcohol, Drugs
Zhu et al. (2018)	China	Patients with MUD	40	32.70 ± 5.27 in the experimental group 35.05 ± 8.02 in the control group	100	Methamphetamine
Khemiri et al. (2019)	Sweden	Patients with AUD	50	49.6 ± 6.1 in active training 49.8 ± 8.7 in control training	50	Alcohol
Rezapour et al. (2019)	Iran	Individuals with opioid use disorder	120	32.26 ± 5.68 in the experimental group 32.30 ± 5.37 in the control group	100	Opioids

*ADS, alcohol dependence syndrome; AUD, alcohol use disorder; M, mean; Protracted methamphetamine, MA; MUD, methamphetamine use disorder; NP, not provided; SD, standard deviation; SUD, substance use disorder*.

#### Date

The studies were published between 1985 and 2019, with two studies published in 2019 (Khemiri et al., 2019; Rezapour et al., 2019), three in 2018 (Gunn et al., 2018; Hendershot et al., 2018; Zhu et al., 2018), three in 2017 (Bell et al., 2017; Brooks et al., 2017; Gamito et al., 2017), three in 2016 (Bell et al., 2016; Brooks et al., 2016; Gamito et al., 2016), two in 2015 (Eack et al., 2015; Rass et al., 2015), two in 1994 (Fals-Stewart and Lucente, 1994; Steingass et al., 1994), and two others in 1985 (Godfrey and Knight, 1985; Godfrey et al., 1985). The remaining studies were published respectively in Gamito et al. (2013, 2014), Rupp et al. (2012), Fals-Stewart and Lam (2010), Goldstein et al. (2005), Peterson et al. (2002), Wetzig and Hardin (1990), Hannon et al. (1989), and Yohman et al. (1988).

#### Country

Twelve of the 26 studies were conducted in the United States (Yohman et al., 1988; Hannon et al., 1989; Wetzig and Hardin, 1990; Fals-Stewart and Lucente, 1994; Peterson et al., 2002; Goldstein et al., 2005; Fals-Stewart and Lam, 2010; Eack et al., 2015; Rass et al., 2015; Bell et al., 2016, 2017; Gunn et al., 2018), four in Portugal (Gamito et al., 2013, 2014, 2016, 2017), two in New Zealand (Godfrey and Knight, 1985; Godfrey et al., 1985), two in South Africa (Brooks et al., 2016, 2017), one in Sweden (Khemiri et al., 2019), one in Iran (Rezapour et al., 2019), one in China (Zhu et al., 2018), one in Canada (Hendershot et al., 2018), one in Austria (Rupp et al., 2012), and one in Germany (Steingass et al., 1994).

#### Age and Gender

Concerning age, nine studies had participants with an average age between 40 and 50 (Yohman et al., 1988; Hannon et al., 1989; Peterson et al., 2002; Rupp et al., 2012; Gamito et al., 2013, 2014, 2016; Rass et al., 2015; Khemiri et al., 2019), seven between 30 and 40 (Wetzig and Hardin, 1990; Fals-Stewart and Lam, 2010; Eack et al., 2015; Gamito et al., 2017; Hendershot et al., 2018; Zhu et al., 2018; Rezapour et al., 2019), four between 20 and 30 (Fals-Stewart and Lucente, 1994; Brooks et al., 2016, 2017; Gunn et al., 2018), and another four between 50 and 60 (Godfrey and Knight, 1985; Steingass et al., 1994; Bell et al., 2016, 2017). In the study conducted by Godfrey et al. (1985), the participants in the experimental group had an average age slightly above 50 and those in the active and passive control groups had an average age slightly above 60. Finally, Goldstein et al. (2005) did not provide information about the age of their participants.

With regards to gender, seven studies had samples exclusively composed of men (Yohman et al., 1988; Hannon et al., 1989; Wetzig and Hardin, 1990; Brooks et al., 2016; Gamito et al., 2017; Zhu et al., 2018; Rezapour et al., 2019), three studies had samples where 90% or above where men (Bell et al., 2016, 2017; Gamito et al., 2016), in five studies men made up between 70 and 90% of the sample (Godfrey et al., 1985; Fals-Stewart and Lucente, 1994; Steingass et al., 1994; Gamito et al., 2013; Eack et al., 2015), and seven studies had more balanced samples with men making up between 40 and 69% of the total participants (Fals-Stewart and Lam, 2010; Rupp et al., 2012; Gamito et al., 2014; Rass et al., 2015; Gunn et al., 2018; Hendershot et al., 2018; Khemiri et al., 2019). Four studies did not present information about the participant's gender distribution (Godfrey and Knight, 1985; Peterson et al., 2002; Goldstein et al., 2005; Brooks et al., 2017). Overall, most studies had samples predominantly constituted by men, with an average of 80% across the 22 studies that presented the necessary data.

#### Substance Type

Regarding the substance type, 14 studies considered only alcohol consumption (Godfrey and Knight, 1985; Godfrey et al., 1985; Yohman et al., 1988; Hannon et al., 1989; Wetzig and Hardin, 1990; Steingass et al., 1994; Peterson et al., 2002; Goldstein et al., 2005; Rupp et al., 2012; Gamito et al., 2013, 2014, 2016; Gunn et al., 2018; Khemiri et al., 2019). Six evaluated the effects of alcohol and other substances (e.g., cannabis, opioids, cocaine; Fals-Stewart and Lucente, 1994; Fals-Stewart and Lam, 2010; Eack et al., 2015; Bell et al., 2016, 2017; Hendershot et al., 2018), three exclusively evaluated methamphetamine-consuming users (Brooks et al., 2016, 2017; Zhu et al., 2018), and three evaluated opioid/methadone users (Rass et al., 2015; Gamito et al., 2017; Rezapour et al., 2019).

### Study Characteristics

The characteristics of the studies (randomization, blindness, control group, and outcome measures) are provided in Table 2.

**Table 2:** Study characteristics.

**References**	**Randomization**	**Blindness**	**Control group (active or passive)**	**Outcome measures**
Godfrey et al. (1985)	Randomized controlled trial	NP	Active	Wechsler Adult Intelligence Scale Wechsler Memory Scale Inpatient Memory Impairment Scale
Godfrey and Knight (1985)	Randomized controlled trial	NP	Active	Wechsler Adult Intelligence Scale Wechsler Memory Scale Inpatient Memory Impairment Scale
Yohman et al. (1988)	Randomized controlled trial	Double-blinded	Passive	Wechsler Memory Scale Wechsler Adult Intelligence Scale Luria Memory Words Test Trail Making Test – Part B
Hannon et al. (1989)	Not randomized controlled trial	NP	Passive	Boston Remote Memory Test Babcock Story Recall Test Hidden Objects Test Memory Matrix Test Rey Auditory Verbal Learning Test
Wetzig and Hardin (1990)	Randomized controlled trial	NP	Active	Adaptation of the Wisconsin Card Sorting Test
Steingass et al. (1994)	Randomized controlled trial	NP	Passive	Reduzierter Wechsler Intelligenztest fur psychiatrische Patienten Mehrfachwahl Wortschatz Test, Version B Wechsler Memory Scale Categorized Verbal Memory Test Color-Word-Association Test Rey Figure Test Street-Map Test D2-Test
Fals-Stewart and Lucente (1994)	Randomized controlled trial	NP	Active	Wechsler Adult Intelligence Scale Tactual Performance Test Trail Making Test – Part B
Peterson et al. (2002)	Randomized controlled trial	Single-blinded	Passive	Wechsler Adult Intelligence Scale Wechsler Memory Scale Trail Making Test – Part B Neuropsychological Assessment Metrics
Goldstein et al. (2005)	Randomized controlled trial	NP	Active	Wechsler Abbreviated Scale of Intelligence Wisconsin Card Sorting Test Trail Making Test Conners Continuous Performance Test
Fals-Stewart and Lam (2010)	Randomized controlled trial	Double-blinded	Active	Neuropsychological Assessment Battery-Screening Module (North American Adult Reading Test)
Rupp et al. (2012)	Randomized controlled trial	NP	Passive	Wechsler Adult Intelligence Scale–Revised Test Battery on Attentional Performance Trail Making Test Munich Verbal Memory Test Complex Figure Test Mehrfachwahl-Wortschatz-Test Multiple-choice vocabulary test designed to measure premorbid (verbal) intelligence Mini-Mental State Examination
Gamito et al. (2013)	Randomized controlled trial	NP	Passive	Mini Mental Examination Test Wisconsin Card Sorting Test Color Trail Test Frontal Assessment Battery
Gamito et al. (2014)	Randomized controlled trial	Single blinded	Passive	Mini Mental State Examination Frontal Assessment Battery Wisconsin Card Sorting Test Color Trail Test
Eack et al. (2015)	Randomized controlled trial	Double-blinded	Passive	NIMH MATRICS Consensus Cognitive Battery
Rass et al. (2015)	Randomized control trial	Double-blinded	Active	Wechsler Adult Intelligence Scale N-back Task Visuo-spatial WM Task Trail Making Test Computerized Digit Symbol Substitution Task Raven's Standard Progressive Matrices Hypothetical Delay Discounting Task The Quick Discounting Operant Task Modified computerized version of the Iowa Gambling Task
Bell et al. (2016)	Randomized controlled trial	Non-blinded	Passive	Mini International Neuropsychiatric Interview Global Assessment of Functioning Wechsler Test of Adult Reading Hopkins Verbal Learning Test Revised
Brooks et al. (2016)	Not randomized controlled trial	NP	Passive	Trail making test
Gamito et al. (2016)	Randomized controlled trial	Double-blinded	Passive	Mini-Mental State Examination Frontal Assessment Battery
Bell et al. (2017)	Randomized controlled trial	NP	Passive	Wechsler Adult Intelligence Scale Mini International Neuropsychiatric Interview Wechsler Test of Adult Reading Continuous Performance Test Trail Making Test – Part A Hopkins Verbal Learning Test Revised Brief Visual Motor Test Wisconsin Card Sorting Test Neuropsychological Assessment Battery Mazes
Brooks et al. (2017)	Randomized controlled trial	NP	Passive	Trail Making Test
Gamito et al. (2017)	Randomized controlled trial	NP	Passive	Mini Mental Examination Test Frontal Assessment Battery Rey Auditory-Verbal Learning Test Semantic Verbal Fluency Task Action Verbal Fluency Task Phonologic Verbal Fluency Task Toulouse Pieron Test's Wisconsin Card Sorting Test Iowa Gambling Task's Color Trails Test's
Gunn et al. (2018)	Randomized controlled trial	NP	Active	Wechsler Abbreviated Scale of Intelligence Near transfer: Rotation Span Reading Span Auditory Consonant Trigram Moderate transfer: Running Letter Span Running Spatial Span Keep Track
Hendershot et al. (2018)	Randomized controlled trial	Double-blinded	Active	Wechsler Adult Intelligence Scale Wechsler Memory Scale
Zhu et al. (2018)	Randomized controlled trial	Double-blinded	Passive	Chinese version of the CogState Battery Delay Discounting Task Iowa Gambling Task Balloon Analog Risk Task
Khemiri et al. (2019)	Randomized control trial	Double-blinded	Active	Wechsler Adult Intelligence Scale Cambridge Neuropsychological Test Automated Battery
Rezapour et al. (2019)	Randomized controlled trial	Single blind	Active	Trial Making Test Digit Span Test Stroop Color-Word Test Verbal Fluency Test Rey Auditory Verbal Learning Test Digit Symbol Substitution Test

*NP, not provided*.

#### Randomization and Blindness

Of the 26 included studies, 22 fall under the category of randomized studies with a control group. Three studies carried out a simple concealment clinical trial (Peterson et al., 2002; Gamito et al., 2014; Rezapour et al., 2019), eight studies were conducted with a double-blind approach (Yohman et al., 1988; Fals-Stewart and Lam, 2010; Eack et al., 2015; Rass et al., 2015; Gamito et al., 2016; Hendershot et al., 2018; Zhu et al., 2018; Khemiri et al., 2019), one mentioned being non-blinded (Bell et al., 2016), and the remaining studies did not provide enough information on the methodology to determine what type of concealment was performed (Godfrey and Knight, 1985; Godfrey et al., 1985; Hannon et al., 1989; Wetzig and Hardin, 1990; Fals-Stewart and Lucente, 1994; Steingass et al., 1994; Goldstein et al., 2005; Rupp et al., 2012; Gamito et al., 2013, 2017; Brooks et al., 2016, 2017; Bell et al., 2017; Gunn et al., 2018).

#### Control Group (Active or Passive)

Of the 26 studies included in the present review, only 11 incorporated an active control group (Godfrey and Knight, 1985; Godfrey et al., 1985; Wetzig and Hardin, 1990; Fals-Stewart and Lucente, 1994; Goldstein et al., 2005; Fals-Stewart and Lam, 2010; Rass et al., 2015; Gunn et al., 2018; Hendershot et al., 2018; Khemiri et al., 2019; Rezapour et al., 2019). The remaining 15 studies used a passive control group (Yohman et al., 1988; Hannon et al., 1989; Steingass et al., 1994; Peterson et al., 2002; Rupp et al., 2012; Gamito et al., 2013, 2014, 2016, 2017; Eack et al., 2015; Bell et al., 2016, 2017; Brooks et al., 2016, 2017; Zhu et al., 2018).

#### Outcome Measures

Regarding the cognitive assessment tools (pre- and post-intervention) used, they varied among the studies, with 11 using the Wechsler Adult Intelligence Scale (Godfrey and Knight, 1985; Godfrey et al., 1985; Yohman et al., 1988; Fals-Stewart and Lucente, 1994; Peterson et al., 2002; Goldstein et al., 2005; Rupp et al., 2012; Rass et al., 2015; Bell et al., 2017; Hendershot et al., 2018; Khemiri et al., 2019), 10 using the Trail Making Test (Yohman et al., 1988; Fals-Stewart and Lucente, 1994; Peterson et al., 2002; Goldstein et al., 2005; Rupp et al., 2012; Rass et al., 2015; Brooks et al., 2016, 2017; Bell et al., 2017; Rezapour et al., 2019), six employing the Wisconsin Sorting Card Test (Wetzig and Hardin, 1990; Goldstein et al., 2005; Gamito et al., 2013, 2014, 2017; Bell et al., 2017), six utilizing the Wechsler Memory Scale (Godfrey and Knight, 1985; Godfrey et al., 1985; Yohman et al., 1988; Steingass et al., 1994; Peterson et al., 2002; Hendershot et al., 2018), five administering the Mini Mental State Examination (Rupp et al., 2012; Gamito et al., 2013, 2014, 2016, 2017), and four using the Frontal Assessment Battery (Gamito et al., 2013, 2014, 2016, 2017). Three studies also used the Iowa Gambling Task (Rass et al., 2015; Gamito et al., 2017; Zhu et al., 2018), and three others the Color Trail Test (Gamito et al., 2013, 2014, 2017). Finally, one study used the Rotation Span (RTS), the Reading Span (RDS), and the Auditory Consonant Trigram (ACT) tasks as near-transfer measures, and the Running Letter Span (RLS), the Running Spatial Span (RSS), and the Keep Track (KT) tasks as moderate-transfer measures (Gunn et al., 2018).

### Characteristics of Interventions

The intervention characteristics (programs, cognitive functions, follow-up, total number of sessions, duration and number of sessions per week, and difficulty level and type of training) are provided in Table 3.

**Table 3:** Intervention characteristics.

**References**	**Intervention details**	**Program**	**Cognitive functions**	**Follow-up**	**Dose^a^**	**Sessions^b^**	**Length^c^**	**Sessions/ week^d^**	**Difficulty level**	**Type of training**
Godfrey et al. (1985)	The memory training program comprised learning and information retrieval tasks, orientation tasks, and exercises to recall recent events for a total of 8 weeks.	Memory training program	Memory	12 months	NP	NP	NP	NP	NP	NP
Godfrey and Knight (1985)	The experimental group performed several memory training taks, with associated learning tasks, Reality Orientation Training, image recognition, and memory retention tasks for recent events.	Memory training program	Memory	14/15 weeks	32 h	32 sessions	1 hour	4 times a week	NP	Manual
Yohman et al. (1988)	The memory training included verbal mediation and also focused on verbal images/materials or “chunking.”	Cognitive training	Multiple skills (learning, memory, problem solving, and perceptual motor)	3 weeks	± 10 h	20 sessions	± 30 min	10 daily sessions	Gradually increased difficulty	NP
Hannon et al. (1989)	The techniques included in the memory program included exercises with visual imagery, attention exercises, external cue strategies, and exercises with verbal strategies.	Memory retraining	Memory	No follow-up	8 h	8 sessions	1 h	Once a week	NP	Manual
Wetzig and Hardin (1990)	A modification of the hierarchical learning intervention designed by Sanders et al. (1975) was used, namely for adults. Thus, the experimental group was provided with a hierarchical cumulative learning program.	Cognitive retraining	Multiple skills (cognitive flexibility, problem solving, and abstract reasoning)	No follow-up	± 90 min	2 sessions	45 min	2 times a week	Gradually increased difficulty	Manual
Steingass et al. (1994)	The intervention consisted of imagery (dual coding) as well as associations of the content. Memory tasks were both verbal and spatial.	Memory training	Attention and memory	No follow-up	± 12 h (training sessions)	12 training sessions / 6 memory-games sessions	1 h (training sessions)	Twice a week training session + daily memory games/scanning tasks	NP	Manual
Fals-Stewart and Lucente (1994)	Thirteen cognitive rehabilitation computer programs were used to remedy cognitive deficits.	Cognitive rehabilitation	Multiple skills (spatial orientation, attention, word memory, and motor)	6 months	± 40 h	48 sessions	50 min	2 times a week	Gradually increased difficulty	Computerized
Peterson et al. (2002)	The NeurXercise^TM^ program consists of several modules in a computerized format to assist individuals who have brain impairment.	NeurXercise^TM^ (computerized cognitive remediation program)	Multiple skills (memory, visuomotor coordination, and visuospatial)	No follow-up	15 h	15 sessions	1 h	NP	Gradually increased difficulty	Computerized
Goldstein et al. (2005)	The cognitive training program included tasks related to spatial abilities, visual scanning, perceptual analysis, concept Formation, and psychomotor speed. The program included tasks of rapid scanning and complex attention.	Version of the Goldman rehabilitation training	Multiple skills (visuospatial abilities, attention, reasoning, and speed of information processing)	No follow-up	7.5 h	15 sessions	30 min	5 times a week	NP	NP
Fals-Stewart and Lam (2010)	PSS CogReHab is a software with four modules that aims to improve the functioning of several cognitive domains. The modules are: foundations, visuospatial, problem solving, and memory.	PSS CogReHab	Multiple skills (attention, memory, executive functioning, visuospatial, and abstract reasoning)	3, 6, 9, and 12 months	± 20 h	24 sessions	50 min	3 times a week	Gradually increased difficulty	Computerized
Rupp et al. (2012)	The Cogpack software includes 62 exercises, each one with 20 alternative variants focused on attention, memory and executive functioning.	Cogpack software	Multiple skills (attention, executive function, and memory domains)	No follow-up	± 12 h	12 sessions	45–60 min	3 times a week	Possible to choose the degree of difficulty	Computerized
Gamito et al. (2013)	The cognitive stimulation program included exercises related to the development of executive functioning skills. Each session consisted of WM, attention, and logical reasoning exercises.	Cognitive stimulation	Multiple skills (attention, WM, and logical reasoning)	No follow-up	12 h	12 sessions	1 h	3 times a week	Gradually increased difficulty	Computerized
Gamito et al. (2014)	The intervention consisted of exercises related to developing executive functioning skills. There were WM, attention, and logical reasoning exercises.	Cognitive stimulation	Executive functioning	After intervention follow-up	10 h	10 sessions	1 h	2/3 times a week	Gradually increased difficulty	Computerized
Eack et al. (2015)	Cognitive Enhancement Therapy is a computer-based training aimed at developing cognitive functions such as memory, attention, and problem solving.	Cognitive Enhancement Therapy	Multiple skills (attention, memory, and problem solving)	No follow-up	60 h	NP	NP	NP	NP	Computerized
Rass et al. (2015)	Cogmed, the WMT program used in this study, included 12 manipulation/maintenance of sequences of information tasks (both verbal and visuo-spatial).	Cogmed QM – WMT	WM	No follow-up	± 18.75 h	25 sessions	45 min	3/5 times a week	Gradually increased difficulty	Computerized
Bell et al. (2016)	Posit Science was used for cognitive training, namely the BrainFitness (auditory) and Insight (visual) sets.	Posit Science – Cognitive Training	Verbal memory and verbal learning	3 and 6 months	± 65 h	65 sessions	1 h	5 times a week	Gradually increased difficulty	Computerized
Brooks et al. (2016)	“Curb Your Addiction (C-Ya)”computerized task, the WMT program used in this study, included up to 20 sessions of a N-back modified task.	“Curb Your Addiction (C-Ya)”computerized task	WM	No follow-up	±10 h	20 sessions	30 min	5 times a week	Gradually increased difficulty	Computerized
Gamito et al. (2016)	The cognitive stimulation program included sessions related to WM, attention and logical reasoning. The tasks progressively increased the level of difficulty.	Cognitive stimulation	Multiple skills (attention, WM, and logical reasoning)	No follow-up	± 8 h	10 sessions	45–50 min	2/3 times a week	Gradually increased difficulty	Computerized
Bell et al. (2017)	Posit Science was used for cognitive training, namely the BrainFitness (auditory) and Insight (visual) sets.	Posit Science – Cognitive Training	Multiple skills (WM and executive functioning)	3 and 6 months	± 65 h	± 65 sessions	1 h	5 times a week	Gradually increased difficulty	Computerized
Brooks et al. (2017)	“Curb Your Addiction (C-Ya)”computerized task, the WMT program used in this study, included up to 20 sessions of a N-back modified task.	“Curb Your Addiction (C-Ya)”computerized task	WM	No follow-up	±10 h	20 sessions	30 min	5 times a week	Gradually increased difficulty	Computerized
Gamito et al. (2017)	The cognitive training program included tasks related to developing executive functioning.	Cognitive training	Executive functioning	End of the treatment follow-up	10 h	10 sessions	1 h	2/3 times a week	Gradually increased difficulty	Computerized
Gunn et al. (2018)	The active WMT consisted of the adaptive Operation Span (OS) and Symmetry Span (SS) tasks.	WMT	WM	30 days	NP	15 sessions	NP	± 3/4 times a week	Gradually increased difficulty	Computerized
Hendershot et al. (2018)	The Cogmed QM program consists of computerized training that includes adaptive memory span tasks in order to contribute to the improvement of WM (verbal and visuospatial).	Cogmed QM 3.0 – Cognitive Training	WM	30 days	± 22.5 h	± 30 sessions	45 min	6 times a week	Gradually increased difficulty	Computerized
Zhu et al. (2018)	The Computerized Cognitive Addiction Therapy includes two attention bias control tasks and two WM tasks.	Mobile-Based Computerized Cognitive Addiction Therapy – Cognitive Rehabilitation	WM	No follow-up	20 h	20 sessions	1 h	5 times a week	Gradually increased difficulty	Computerized
Khemiri et al. (2019)	Each cognitive training session comprised eight verbal and visuospatial WM exercises.	Cogmed – Cognitive Training	WM	Weekly follow-up	10/18 h	20/25 sessions	30/45 min	5 times a week	Gradually increased difficulty	Computerized
Rezapour et al. (2019)	The cognitive rehabilitation program used was NECOREDA. This is a pencil-and-paper extension, developed for the rehabilitation of the main cognitive functions affected in substance use disorder. Also included are concepts of psychoeducation in cognitive rehabilitation.	NEuroCOgnitiveREhabilitation for Disease of Addiction program (NECOREDA) – Cognitive Rehabilitation	Multiple skills (attention, WM, visuospatial process, verbal skills, and executive functions)	1, 3, and 6 months	16 h	16 sessions	1 h	2 times a week	Gradually increased difficulty	Manual

a *Total number of training/stimulation/rehabilitation hours*.

b *Total number of cognitive training/stimulation/rehabilitation sessions*.

c *Session length (minutes)*.

d *Number of sessions per week*.

*NP, not provided*.

#### Programs

Eighteen of the 26 studies considered their intervention to be a cognitive training program (Godfrey and Knight, 1985; Godfrey et al., 1985; Yohman et al., 1988; Hannon et al., 1989; Wetzig and Hardin, 1990; Steingass et al., 1994; Peterson et al., 2002; Goldstein et al., 2005; Eack et al., 2015; Rass et al., 2015; Bell et al., 2016, 2017; Brooks et al., 2016, 2017; Gamito et al., 2017; Gunn et al., 2018; Hendershot et al., 2018; Khemiri et al., 2019), five considered their intervention to be a cognitive rehabilitation program (Fals-Stewart and Lucente, 1994; Fals-Stewart and Lam, 2010; Rupp et al., 2012; Zhu et al., 2018; Rezapour et al., 2019), and the remaining three considered it to be a cognitive stimulation program (Gamito et al., 2013, 2014, 2016; Table 3).

#### Cognitive Functions

The targeted cognitive domains also varied across studies. Twelve studies evaluated more than two cognitive skills (e.g., attention, memory, problem solving, abstract reasoning, processing speed, etc.; Yohman et al., 1988; Wetzig and Hardin, 1990; Fals-Stewart and Lucente, 1994; Peterson et al., 2002; Goldstein et al., 2005; Fals-Stewart and Lam, 2010; Rupp et al., 2012; Gamito et al., 2013, 2016; Eack et al., 2015; Bell et al., 2017; Rezapour et al., 2019). Ten studies assessed memory (Godfrey and Knight, 1985; Godfrey et al., 1985; Hannon et al., 1989; Rass et al., 2015; Brooks et al., 2016, 2017; Gunn et al., 2018; Hendershot et al., 2018; Zhu et al., 2018; Khemiri et al., 2019), with seven focusing on WM (Rass et al., 2015; Brooks et al., 2016, 2017; Gunn et al., 2018; Hendershot et al., 2018; Zhu et al., 2018; Khemiri et al., 2019), two assessed executive functions (Gamito et al., 2014, 2017), one assessed attention and memory (Steingass et al., 1994), and one assessed verbal memory and verbal learning (Bell et al., 2016; Table 3). Only four studies evaluated processing speed (Gamito et al., 2014; Eack et al., 2015; Bell et al., 2017; Rezapour et al., 2019).

#### Follow-Up

Of the 26 studies analyzed, 13 reported a follow-up (Godfrey and Knight, 1985; Godfrey et al., 1985; Yohman et al., 1988; Fals-Stewart and Lucente, 1994; Fals-Stewart and Lam, 2010; Gamito et al., 2014, 2017; Bell et al., 2016, 2017; Gunn et al., 2018; Hendershot et al., 2018; Khemiri et al., 2019; Rezapour et al., 2019). Regarding the period of time during which the follow-up took place, there were differences among the studies, with the follow-up taking place 3 weeks to 1 year after the treatment. In three studies, follow-up occurred shortly after the end of the intervention (Gamito et al., 2014, 2017; Khemiri et al., 2019).

#### Total Number of Sessions, Duration, and Number of Sessions per Week

Table 3 presents the total number, duration, and the number of sessions per week. With regard to computer programs, three studies used the program Cogmed, two of which held 25 sessions (30/45 min, 3/5 times a week; Rass et al., 2015; Khemiri et al., 2019), while the other held ~30 sessions (45 min, six times a week; Hendershot et al., 2018). Two studies employed the Posit Science program, having carried out ~65 sessions lasting 1 h for five times a week (Bell et al., 2016, 2017). Two studies used a computer-based WM task called “Curb Your Addiction (C -Ya)” and held up to 20 sessions (30 min, five times a week; Brooks et al., 2016, 2017). One study used the PSS CogRehab program, with 24 sessions (50 min, three times a week; Fals-Stewart and Lam, 2010). Another study used the Cogpack software over 12 sessions (45–60 min, three times a week; Rupp et al., 2012). Other computer programs were also used, namely NEuroCOnitiveREhabilitation for Disease of Addiction (NECOREDA; Rezapour et al., 2019), Mobile-Based Computerized Cognitive Addiction Therapy (CCAT; Zhu et al., 2018), and NeurXerciseTM (Peterson et al., 2002). These programs were applied with 1 hour sessions; they varied only in the total number of sessions (16, 20, and 15, respectively). Four studies used Unity 2.5 technology to develop their programs. These consisted of ~10–12 sessions (Gamito et al., 2013, 2014, 2016, 2017). In each of these studies, the sessions took 1 h 2–3 times a week (Gamito et al., 2013, 2014, 2017) or 45/50 min 2–3 times a week (Gamito et al., 2016).

One study considered modification of the hierarchical learning intervention (two sessions of 45 min; Wetzig and Hardin, 1990), one used the adaptive Operation Span (OS) and Symmetry Span (SS) tasks in 15 training sessions over <4 weeks (Gunn et al., 2018), one resorted to a cognitive training program of ~12 training sessions and six memory-game sessions (twice a week training sessions and daily memory games/scanning tasks; Steingass et al., 1994), another applied a Cognitive Enhancement Therapy, with 60 h of training (Eack et al., 2015), and another included the version of the Goldman rehabilitation training (15 sessions of 30 min each, five times a week; Goldstein et al., 2005). One study used a cognitive training program, however the authors did not provide details on the intervention (Godfrey et al., 1985), and another study employed a cognitive rehabilitation program of 48 sessions (50 min each, twice a week; Fals-Stewart and Lucente, 1994).

There were also two studies that used specific memory training programs: one included a 32-session memory training program (1 h, four times a week; Godfrey and Knight, 1985), and the other a retraining memory program consisting on eight 1-h sessions (Hannon et al., 1989). Finally, Yohman et al. (1988) used a cognitive training program consisting of 20 sessions of ~30 min each. Considering the 24 studies that provided information on the number of sessions, there was an average of ~20 training sessions per intervention. However, from the total 18 studies that found some kind of cognitive improvement resulting from the cognitive training, the average number of sessions was slightly superior, at 23 sessions per intervention. The details of the interventions can be found in more detail in Table 3.

#### Difficulty Level

Considering the difficulty levels of the intervention tasks, 19 studies chose to gradually increase the degree of difficulty throughout the intervention, starting with simpler task sessions and gradually introducing more complex task sessions (Yohman et al., 1988; Wetzig and Hardin, 1990; Fals-Stewart and Lucente, 1994; Peterson et al., 2002; Fals-Stewart and Lam, 2010; Gamito et al., 2013, 2014, 2016, 2017; Rass et al., 2015; Bell et al., 2016, 2017; Brooks et al., 2016, 2017; Gunn et al., 2018; Hendershot et al., 2018; Zhu et al., 2018; Khemiri et al., 2019; Rezapour et al., 2019). One study indicated that in each exercise it was possible to choose the degree of difficulty (Rupp et al., 2012). Only six studies did not mention anything about this topic (Godfrey and Knight, 1985; Godfrey et al., 1985; Hannon et al., 1989; Steingass et al., 1994; Goldstein et al., 2005; Eack et al., 2015).

#### Types of Training

With regard to the type of training, only five studies used paper-and-pencil training (Godfrey and Knight, 1985; Hannon et al., 1989; Wetzig and Hardin, 1990; Steingass et al., 1994; Rezapour et al., 2019). Three studies did not mention the specific training type (Godfrey et al., 1985; Yohman et al., 1988; Goldstein et al., 2005). The remaining 18 studies used computerized training (Fals-Stewart and Lucente, 1994; Peterson et al., 2002; Fals-Stewart and Lam, 2010; Rupp et al., 2012; Gamito et al., 2013, 2014, 2016, 2017; Eack et al., 2015; Rass et al., 2015; Bell et al., 2016, 2017; Brooks et al., 2016, 2017; Gunn et al., 2018; Hendershot et al., 2018; Zhu et al., 2018; Khemiri et al., 2019).

### Key Findings

Table 4 describes the main results for the 26 studies included in the review. In 16 of the 26 studies, the authors reported clear significant cognitive improvements in individuals who received cognitive training (Wetzig and Hardin, 1990; Fals-Stewart and Lucente, 1994; Steingass et al., 1994; Goldstein et al., 2005; Fals-Stewart and Lam, 2010; Rupp et al., 2012; Gamito et al., 2013, 2014, 2016, 2017; Bell et al., 2016, 2017; Gunn et al., 2018; Zhu et al., 2018; Khemiri et al., 2019; Rezapour et al., 2019). In another two studies, the authors reported marginally significant cognitive improvements (Yohman et al., 1988; Hendershot et al., 2018). From the total 18 studies that found some kind of cognitive improvement, two (Yohman et al., 1988; Khemiri et al., 2019), reported that they were not found in all the assessed cognitive functions. Yohman et al. (1988) reported that although the memory-training group showed no significant improvements in memory tests, the problem-solving group showed marginally significant improvements in problem-solving tests. Khemiri et al. (2019) indicated that there was no enhancement in visuospatial WM however there was a significant increase in the verbal WM ability.

**Table 4:** Key findings.

**References**	**Study aim(s)**	**Key finding(s)**
Godfrey et al. (1985)	Evaluate long-term memory improvements in participants having as a base an intensive memory rehabilitation program for amnesic alcoholics	Both the memory training group and the active control group showed improved memory function in the post-test. There is no information about a statistical comparison between the groups in order to examine possible differences.
Godfrey and Knight (1985)	Understand whether the memory function can be generalized to other memory functioning tasks and determine the duration of maintenance of the gains in question	The control group showed the same benefits in memory performance as the experimental group.
Yohman et al. (1988)	Determine whether the neuropsychological areas involved in patients with alcoholism who undergo cognitive training have improved compared with individuals who have not received any type of training; understand whether other cognitive areas can benefit from training, even if it is specific to a certain area	The problem-solving group showed improvements in the results of the problem-solving tests compared with the group that did not receive any training. However, the problem-solving group did not show increase in terms of memory and in perceptual-motor skills.
Hannon et al. (1989)	Examine the effectiveness of memory retraining in individuals with alcohol problems	The results did not show sufficient support to confirm the objective of the study. Only the Memory Matrix Test showed gains between the pre- and the post-test.
Wetzig and Hardin (1990)	Understand whether cognitive retraining impacts a sample of individuals with SUD and cognitive impairment	Individuals who received remedial training achieved an equal and superior performance on the Wisconsin Card Sorting Test than the general population.
Steingass et al. (1994)	Determine whether semantically encoded material is favored by the treatment	The experimental group that received treatment showed improvements in terms of reproduction of figures and verbal memory.
Fals-Stewart and Lucente (1994)	Based on a cognitive rehabilitation program, evaluate whether there are neuropsychological changes in a sample of individuals with drug use and the presence of cognitive deficits	During the first 2 months of treatment, patients who received the cognitive rehabilitation program showed gains in cognitive functioning: Cerebral recovery was faster in these patients.
Peterson et al. (2002)	Investigate the efficacy of the NeurXerciseTM program, which concerns a computerized cognitive remediation program, within the scope of cognitive recovery	The effectiveness of the computerized cognitive remediation program used in the study was not confirmed. There were no statistically significant differences between the group that received the program, the placebo group, and the group without intervention.
Goldstein et al. (2005)	Investigate the effectiveness of a cognitive training program in order to benefit the cognitive functioning of individuals with alcohol use disorder and comorbidities with other neuropsychiatric disorders, namely in the subacute phase of detoxification	There were cognitive increases in the experimental group compared to the placebo group, namely in the conceptual flexibility and attention.
Fals-Stewart and Lam (2010)	Evaluate whether patients in the experimental group who received standard treatment plus computer-assisted cognitive rehabilitation, compared with a control group who received an intensive care program, showed better results in cognitive functioning	The group with standard treatment plus computer-assisted cognitive rehabilitation showed a faster overall improvement in cognitive functioning compared to the control group. However, it was not possible to determine whether these improvements were differential for the various cognitive functions.
Rupp et al. (2012)	Assess whether cognitive remediation therapy during treatment improves cognitive functioning in patients with alcohol use disorder.	The group that received cognitive remediation therapy showed significant increment in memory, executive functioning and care, especially in WM delayed memory and attention (divided attention and alertness). Improvements were also noted in the Mini Mental State Examination and Complex Figure Test indices.
Gamito et al. (2013)	Evaluate the effect of cognitive stimulation using serious games in a sample of patients with alcohol dependence syndrome	There were improvements in the general cognitive functions assessed in all groups. However, there was an improvement in the frontal area in the cognitive functioning of the individuals in the group who received a cognitive stimulation program, using mobile technology.
Gamito et al. (2014)	Evaluate the cognitive effects in a sample of individuals with alcohol dependence based on a neuropsychological intervention using serious games and mobile technology	There was an increase in general cognitive skills, both in the control group and in the experimental group. However, the improvement was more significant in terms of frontal lobe functions in the experimental group. Processing speed was evaluated using two versions of the Color Trail Test (CTT). Although there was a decrease in the error rate and execution time of CTT1 and CTT2, there was no statistically significant interaction in terms of the treatment factor.
Eack et al. (2015)	Evaluate the efficacy and feasibility of using Cognitive Enhancement Therapy in a sample of patients with schizophrenia and alcohol/cannabis misuse	Cognitive Enhancement Therapy was an effective and viable treatment for cognitive impairments in schizophrenic patients with alcohol/cannabis problems. The neurocognitive gains were most evident in verbal learning and processing speed (NIMH MATRICS Consensus Cognitive Battery), although neither showed statistically significant differences.
Rass et al. (2015)	Examine whether WMT brings cognitive changes in a sample of methadone maintenance patients.	The experimental group of methadone maintenance patients achieved improvements in some measures of WM after receiving WMT, namely in visuospatial WM and digit span. However, there were no improvements on WM measures dissimilar from the training tasks.
Bell et al. (2016)	Evaluate the efficacy of cognitive training in memory deficits and verbal learning of older veterans with alcohol use disorder	Cognitive training in conjunction with work therapy was effective in ameliorating memory deficits in a sample of individuals with alcohol use disorder.
Brooks et al. (2016)	Evaluate the effect of standard psychological TAU and adjunct WMT on brain volume in male in-patients receiving treatment for methamphetamine (MA) use.	The control group (TAU) presented larger volume in the bilateral putamen and reduced volume in the left middle temporal gyrus, right post-central gyrus and left insula cortex. The experiemntal group (TAU + WMT) showed more pronounced increases in volume that extended across large areas of the bilateral basal ganglia, along reduced bilateral cerebellar volume. WM accuracy at post-test in the experimental group was associated with larger volume in the right middle frontal cortex and orbitofrontal cortex. While there was an improvement in WM accuracy in the experimental group, no near-transfer effects were found (no changes in the Trail Making Test).
Gamito et al. (2016)	Evaluate the efficacy of a Cognitive Stimulation Program, using mobile devices, related to the cognitive rehabilitation of recovering alcoholic individuals	There was significant benefit in terms of frontal lobe functioning in the experimental group.
Bell et al. (2017)	Test whether the group of individuals who received cognitive remediation therapy and work therapy showed improvements in neurocognitive functions compared with a group that only received work therapy	There were significant differences in the executive functioning indexes in the group that received cognitive remediation therapy and work therapy. There were no statistically significant differences in the rate of change of processing speed between cognitive remediation therapy with work therapy and the work therapy with treatment as usual.
Brooks et al. (2017)	Evaluate the impact of daily WMT alongside treatment as usual (TAU) on self-report measures of impulsivity and self regulation in patients receiving treatment for methamphetamine (MA) use.	From the experimental group (TAU + WMT), those who engaged in the highest level of training had a learning effect of 35% between pre and post-test, and showed significant changes in self-reported impulsivity and self-regulation scores. There were no significant differences in executive measures (Trail Making Test) between pre and pot-test in the experimental group.
Gamito et al. (2017)	Analyze the efficacy of cognitive training in the rehabilitation and stimulation of addicts in recovery, based on a serious games approach	There was an increase in cognitive functioning in terms of frontal brain functions as well as sustained attention and verbal memory. There were also improvements in decision-making and cognitive flexibility.
Gunn et al. (2018)	Examine the efficacy a complex WMT program in those with an alcohol use disorder (AUD), as well as predictors of training improvement.	There was significant transfer on two near WM transfer measures (Rotation Span and Auditory Consonant Trigram) at post-test and 30-day follow-up for individuals who completed the WMT, independent of the group (AUD vs. healthy control). There was also evidence of transfer on one moderate transfer task (Running Spatial Span) at post-test, but not on the 30-day follow-up.
Hendershot et al. (2018)	Assess whether the WMT together with treatment as usual contributes to improvements in executive functioning in the short term	There were marginally significant improvements found in the digit span (primary outcome) and in the results of the Cogmed Progress Indicator index (secondary outcome). There were no other secondary outcome improvements to support the efficacy of WMT.
Zhu et al. (2018)	Understand whether cognitive impairments can be improved based on the Computerized Cognitive Addiction Therapy (CCAT) application	Comparing with the control group, the CCAT group had better cognitive performance after 4 weeks of training as well as better performance on impulsive control tasks.
Khemiri et al. (2019)	Test the efficacy and viability of a WMT program (computerized) in patients with alcohol use disorder	The experimental group saw significant improvements in verbal, but not spacial, WM functioning. No effect of WMT was found on other cognitive functions.
Rezapour et al. (2019)	Evaluate the efficacy of a cognitive rehabilitation treatment with a view to improving the neurocognitive functions of individuals with opioid use disorder	The group of individuals who received cognitive rehabilitation treatment showed significant improvements in terms of processing speed, WM, and memory span. There was also an increase in these individuals in the switching and learning tests. In turn, these effects were shown to persist for at least 6 months.

Two studies presented somewhat ambiguous results. One study (Eack et al., 2015) reported significant improvement in neurocognition, but described the differences in the areas where the biggest changes were found (processing speed and verbal learning) as failing traditional significant thresholds. Another (Godfrey et al., 1985), reported significant improvements in memory functioning for both the training and active control groups, without presenting data on the statistical comparison between them.

One study (Rass et al., 2015) discriminated results regarding similar and dissimilar measures to the training tasks, reporting improvements in some measures of WM (visuospatial WM and digit span) similar to the training tasks, although no improvements in their dissimilar equivalent.

Finally, the efficacy/effectiveness of cognitive training was not supported in five studies (Godfrey and Knight, 1985; Hannon et al., 1989; Peterson et al., 2002; Brooks et al., 2016, 2017). Godfrey and Knight (1985) reported that the control and experimental groups showed the same improvement in terms of memory functioning. Hannon et al. (1989) concluded that the obtained results did not show sufficient support to confirm the objective of the study. However, there was still an increase in the Memory Matrix Test between the pre-test and the post-test. Peterson et al. (2002) did not confirm the efficacy of the Computerized Cognitive remediation program. Brooks et al. (2016) found that WM accuracy was improved in the experimental group, but that no near-transfer effects were found (no significant differences in the Trail Making Test). However, the experimental group did show more pronounced neural changes. Similarly, Brooks et al. (2017) reported a learning effect of 35% between pre and post-test, but no significant differences in executive measures (Trail Making Test).

### Risk of Bias

In the present literature review, the risk of bias in randomized controlled trials was assessed using the Cochrane Risk of Bias Tool (Higgins et al., 2011). In turn, the non-randomized study (Hannon et al., 1989) was assessed for risk of bias using the ROBINS-I tool (Sterne et al., 2016). Since the methodological details of many of the studies included in the present review were incomplete or not sufficiently detailed (see Supplementary Material), we consider that the risk-of-bias assessment has limitations. However, we observed that the most common possible sources of bias in the randomized controlled trials studies selected for this review refers to the blinding of participants and personnel (performance bias) and blinding of outcome assessment (detection bias). There were also 14 studies in which we were unable to assess the type of concealment performed (Godfrey and Knight, 1985; Godfrey et al., 1985; Yohman et al., 1988; Wetzig and Hardin, 1990; Fals-Stewart and Lucente, 1994; Steingass et al., 1994; Peterson et al., 2002; Goldstein et al., 2005; Fals-Stewart and Lam, 2010; Rupp et al., 2012; Gamito et al., 2013, 2014; Eack et al., 2015; Brooks et al., 2017; Gunn et al., 2018) due to the lack of methodological information (as can be seen Supplementary Material). This lack of information is also a possible source of bias. With reference to low risk of bias, after complete analysis, only two studies (Hendershot et al., 2018; Khemiri et al., 2019) presented a low risk of bias in all the assessment domains (see Supplementary Material).

On the other hand, the quasi-experimental study included (Hannon et al., 1989) in the present review presented a moderated risk of bias on the baseline confounding, selection of participants and selection of reported results. There were also domains (deviation from intended information and missing data) where there can be possible risk of bias due to lack of information provided (see Supplementary Material).

The presented final assessment was discussed between the two reviewers (TC; CC) who examined the discrepancies between the performed evaluations. In situations where the reviewers did not reach a consensus, a third reviewer intervened (TA). The data found highlights selection bias, performance bias and detection bias as risk of bias for the cumulative evidence for the present review.

## Discussion

The main goal of the present review was to understand what the state of the art tells us with reference to the effectiveness of cognitive training interventions in improving memory and/or executive functioning in individuals with SUD. Although this review will certainly not resolve the controversy regarding cognitive training, we hope that it will serve as a pertinent contribution to what is, without a doubt, a very important debate.

The majority of the reviewed studies showed either clear (Wetzig and Hardin, 1990; Fals-Stewart and Lucente, 1994; Steingass et al., 1994; Goldstein et al., 2005; Fals-Stewart and Lam, 2010; Rupp et al., 2012; Gamito et al., 2013, 2014, 2016, 2017; Bell et al., 2016, 2017; Gunn et al., 2018; Zhu et al., 2018; Khemiri et al., 2019; Rezapour et al., 2019) or marginally significant (Yohman et al., 1988; Hendershot et al., 2018) improvements on at least one of the cognitive domains considered, giving strength to the hypothesis that cognitive training can be a relevant addition to SUD treatment. Moreover, even though that was not the focus of this review, it is important to note that various studies (even some that did not see significant cognitive improvements; Fals-Stewart and Lucente, 1994; Fals-Stewart and Lam, 2010; Rupp et al., 2012; Eack et al., 2015; Rass et al., 2015; Brooks et al., 2016, 2017; Rezapour et al., 2019) reported a positive impact of cognitive training on clinical and/or SUD variables.

### The Impact of Cognitive Training on Memory in SUD

From all cognitive domains, memory was the domain most targeted in the reviewed studies. This is likely explained by the fact that memory is not only one of the areas most affected by substance use, but also one believed to impact treatment outcomes.

Significant improvements regarding memory could be found in studies with various SUD populations (i.e., substance of use). When considering overall memory capacity, positive and significant effects were found in participants who consumed both alcohol and other substances (Bell et al., 2016). Concerning WM specifically, significant improvements were shown for both participants who consumed only alcohol (Rupp et al., 2012; Gunn et al., 2018; Khemiri et al., 2019), and those who also used other substances (Hendershot et al., 2018). Khemiri et al. (2019) discriminated between verbal and visuospatial WM, and only found significant changes for the first. Delayed and verbal memory were also studied subdomains, and positive changes in these were found in alcohol-consuming participants (Rupp et al., 2012), and opioid-consuming participants (Gamito et al., 2017).

It is also important to analyse the studies that did not found significant memory improvements following cognitive training. From the studies that showed a clear lack of cognitive improvement after cognitive training, four studies focused on memory (Godfrey and Knight, 1985; Hannon et al., 1989; Brooks et al., 2016, 2017) with two of those specifically on WM (Brooks et al., 2016, 2017), and one considered a number of cognitive functions (e.g., visual-motor coordination, visual-spatial skills) including memory (Peterson et al., 2002). Regarding population, three of these studies explored the effectiveness of cognitive training in alcoholics (Godfrey and Knight, 1985; Hannon et al., 1989; Peterson et al., 2002), and two in methamphetamine users (Brooks et al., 2016, 2017).

Some of these studies presented significant limitations that may have affected the results, such as small sample size and/or high drop-out rate (Godfrey and Knight, 1985; Peterson et al., 2002), reported possible insensitivity of outcome measures (Godfrey and Knight, 1985; Hannon et al., 1989), and a lack of specificity in the training techniques (Godfrey and Knight, 1985). Moreover, Peterson et al. (2002), proposed that the lack of baseline cognitive impairment in their study participants may explain theirs result. They pointed out that cognitive training may be more effective on those with at least mild to moderate baseline cognitive impairment, something that would be interesting to consider in future research. Interestingly, two of these studies (Godfrey and Knight, 1985; Hannon et al., 1989) delivered the cognitive training intervention in a group setting.

Rass et al. (2015) and Brooks et al. (2016, 2017), presented results that justify a more in-depth look. Rass et al. (2015) had the only study that clearly discriminated results according to the measures' level of similarity to the training tasks. They found that there were significant improvements in some measures of WM similar to the training tasks, but no improvements in dissimilar measures. These results indicate the presence of “near” but not “far transfer” effects, and highlight the root of the on-going debate about cognitive training effectiveness. Brooks et al. (2016) too found that although WMT did not lead to significant changes in the cognitive measures used (i.e., Trail Making Test), it did increase memory accuracy (in the training tasks). In turn, memory accuracy showed itself to be connected with larger volume in the right middle frontal cortex and orbitofrontal cortex, both regions associated with WM ability and executive functioning. Brooks et al. (2017), found similarly that WMT did not lead to significant improvements in the cognitive measures used (i.e., Trail Making Test), but did lead to a learning effect of 35% and significant changes in self-report measures looking into impulsivity and self-regulation. These results are intriguing and bring up questions about the efficacy of cognitive measures in evaluating potential benefits of WMT, or cognitive training in general, and in adequately assessing “far transfer” effects. In a more recent review study, Brooks et al. (2020) reported that WMT can lead to significant neural effects often in the absence of behavioral changes. Moreover, various neuroimaging studies appeared to have found “far transfer” effects of WMT to other un-related cognitive domains, something that might be harder to measure.

### The Impact of Cognitive Training on Executive Functioning and Processing Speed in SUD

Similarly to memory, executive functioning was also studied in different SUD populations (i.e., substance of use). Bell et al. (2017) found significant improvements on neurocognitive measures of executive functioning in participants who consumed both alcohol and other substances following 13 weeks (5 h/week) of cognitive training (both auditory and visual tasks). In line with these findings, Gamito et al. (2017) showed an improvement on the frontal lobe functions of opioid-consuming participants after 10 cognitive training sessions. Concerning mental flexibility specifically, significant improvements were found in alcohol-consuming participants (Gamito et al., 2014). Finally, problem-solving, which is a skill strongly associated with executive functioning, also showed significant positive effects in the same population (Yohman et al., 1988).

In comparison with memory and executive functions, there appears to be a lack of interest in studying the impact of cognitive training on processing speed. From the studies included in the review, only four targeted this cognitive domain (Gamito et al., 2014; Eack et al., 2015; Bell et al., 2017; Rezapour et al., 2019). And, from those, only Rezapour et al. (2019) reported significant improvements in the processing speed of individuals with opioid use disorder who received cognitive training. These improvements persisting for at least 6 months.

### Cognitive Training Programs

Cognitive training programs have suffered significant changes over the years as a result of technological advancement. When these programs first started to be used, they were administered with a paper-and-pencil modality, but today most new cognitive training programs created are computer- or even mobile-based. The studies included in this review reflected this tendency, with the majority of cognitive training programs used being computerized (Fals-Stewart and Lucente, 1994; Peterson et al., 2002; Fals-Stewart and Lam, 2010; Rupp et al., 2012; Gamito et al., 2013, 2014, 2016, 2017; Eack et al., 2015; Rass et al., 2015; Bell et al., 2016, 2017; Brooks et al., 2016, 2017; Gunn et al., 2018; Hendershot et al., 2018; Zhu et al., 2018; Khemiri et al., 2019).

Among the studies that used computerized programs, many created unique training regimes by adapting relevant cognitive tasks, while some used already recognized cognitive training programs. Cogmed was the most used program (Rass et al., 2015; Hendershot et al., 2018; Khemiri et al., 2019), followed by Posit Science (Bell et al., 2016, 2017), and the computer-based WM training program “Curb Your Addiction (C- Ya)” (Brooks et al., 2016, 2017). Other computerized training programs used were PSS CogRehab (Fals-Stewart and Lam, 2010), Cogpack (Rupp et al., 2012), NeurXerciseTM (Peterson et al., 2002), and Mobile-Based Computerized Cognitive Addiction Therapy (CCAT; Zhu et al., 2018).

Only five studies declared using paper-and-pencil training programs, and predictably four of those were among the oldest studies included in the review (Godfrey and Knight, 1985; Hannon et al., 1989; Wetzig and Hardin, 1990; Steingass et al., 1994). Interestingly, the fifth study (Rezapour et al., 2019), used the recently developed paper and pencil cognitive rehabilitation package NEuroCOnitiveREhabilitation for Disease of Addiction (NECOREDA).

There is another, more recent, type of cognitive training intervention that we did not considered in this review for lack of any studies that met the inclusion criteria-Virtual Reality programs. These type of interventions have shown promising results in other diseases and/or disorders that involve impairment of cognitive functions (Pedroli et al., 2018). However, to date, most studies that use virtual reality in the scope of SUD seek to understand the relationship between environmental stimuli and drug use (Bordnick et al., 2011; Hone-Blanchet et al., 2014). Indeed, studies that explore virtual reality as a cognitive training tool in SUD are scarce. To our knowledge, only Man (2018) has studied the effectiveness of this type of intervention on the improvement of cognitive functioning in individuals with substance abuse disorders. The results appear promising. As a drastically different form of delivering cognitive training, it is important that more research be conducted to study its effectiveness and compare it to the type of interventions used to date.

### Limitations

The presented results need to be interpreted taking into account this review's limitations. Of the 26 studies presented, 15 did not have an active control group (Yohman et al., 1988; Hannon et al., 1989; Steingass et al., 1994; Peterson et al., 2002; Rupp et al., 2012; Gamito et al., 2013, 2014, 2016, 2017; Eack et al., 2015; Bell et al., 2016, 2017; Brooks et al., 2016, 2017; Zhu et al., 2018), and 13 did not have a follow-up (Hannon et al., 1989; Wetzig and Hardin, 1990; Steingass et al., 1994; Peterson et al., 2002; Goldstein et al., 2005; Rupp et al., 2012; Gamito et al., 2013, 2016; Eack et al., 2015; Rass et al., 2015; Brooks et al., 2016, 2017; Zhu et al., 2018). There were also three studies that showed only a follow-up right after the intervention (Gamito et al., 2014, 2017; Khemiri et al., 2019). These limitations make it impossible to effectively account for possible placebo effects as well as infer the maintenance of any real effects over time.

It is also important to highlight the diversity of cognitive training programs (e.g., administration, duration, number of sessions, and hours of training) and populations (i.e., substance of use, time of abstinence) included in the reviewed studies. This heterogeneity, along with the lack of detailed information about the used interventions found in many studies, prevented us from analyzing the results more in-depth and from evaluating the real impact of these variables, for example on effect size. It also made it impossible to generalize about the improvements obtained in cognitive functions for the general population with SUD.

Finally, the lack of concealment concerning the researchers in most of the included studies in the present review stands out, along with the fact that some studies failed to provide information regarding the methodology used for concealment of the participants.

## Conclusions

Overall, this review found that the majority of the included studies reported cognitive improvements following cognitive training, including in two of our main domains of interest-memory and executive functioning. In addition, various studies also found that cognitive training led to significant changes in clinical (e.g., treatment engagement) and SUD variables (e.g., substance use, relapse rate), even though the mechanisms behind these improvements are not completely understood.

Although the results appear promising, the heterogeneity among the studies regarding the type of cognitive training program used and the population studied demands further and more careful research. To this end, future studies should explore the comparative effectiveness of similar cognitive training programs on different SUD populations. Moreover, they should also study the impact of structural variables (such overall duration, number of sessions, and hours of training), on the effectiveness of the programs. This data would be relevant to understand the feasibility (and cost-benefit) of integrating these type of interventions in different clinical settings.

Concerning the controversy about the generalization (or lack thereof) of cognitive gains from cognitive training, we support those who have suggested that many of the studies conducted to date have been too narrow in their approach. We believe future research into cognitive training effectiveness may gain from broadening the concepts of “far-transfer,” as well as from considering multiple forms of assessment (e.g., cognitive tests, neuroimaging, and self-report questionnaires) when measuring potential effects.

It is becoming clear that, if we want to bring clarity to the discussion surrounding the effectiveness of cognitive training, we should not only start asking more nuanced questions, but also considering that the answers may likewise be more complex.

## Data Availability Statement

The original contributions presented in the study are included in the article/Supplementary Material, further inquiries can be directed to the corresponding author/s.

## Author Contributions

TC, MP, and MD contributed to the conception and design of the study, constant revision, wrote the article, which was critically revised by all the other authors, and revised the manuscript critically for relevant intellectual content. TC and ER conducted the literature search, selection, data extraction, and analysis. TC and CC conducted the assessment of study quality. Disagreements were resolved by TA. TC, ER, and CC revised the last version of the manuscript. All authors contributed to manuscript revision, read, and approved the submitted version.

## Conflict of Interest

ER is the Executive and Clinical Director of VillaRamadas International Treatment Center, an institution that provides addiction treatment. Even though the intervention analyzed in this systematic review (cognitive training) does not feature in the center's current therapeutic program, there is the intention of studying its effectiveness with the center's population in the future. The remaining authors declare that the research was conducted in the absence of any commercial or financial relationships that could be construed as a potential conflict of interest.

## Publisher's Note

All claims expressed in this article are solely those of the authors and do not necessarily represent those of their affiliated organizations, or those of the publisher, the editors and the reviewers. Any product that may be evaluated in this article, or claim that may be made by its manufacturer, is not guaranteed or endorsed by the publisher.

## Abbreviations

JBI: Joanna Briggs Institute
PRISMA: Preferred Reporting Items for Systematic Reviews and Meta-Analyses
ROBINS-I: Risk of Bias in Non-randomized Studies - of Interventions
SUD: Substance Use Disorders
WMT: Working Memory Training
WM: Working Memory.
