# Supplementary material for: Cognitive Training Effectiveness on Memory, Executive Functioning, and Processing Speed in Individuals With Substance Use Disorders: A Systematic Review
Source: Front Psychol. 2021 Aug 13;12:730165. doi: 10.3389/fpsyg.2021.730165 (PMC8418081; doi:10.3389/fpsyg.2021.730165)
Supplement: Supplementary file 1 [file Table_1.DOCX]

Supplementary Material. Risk of Bias

**S1.** *Risk-of-bias Assessment of the Randomized Controlled Trials*

| Author (Year) | Random sequence generation (selection bias) | Allocation concealment (selection bias) | Blinding of participants and personnel (performance bias) | Blinding of outcome assessment (detection  bias) | Incomplete outcome data (attrition bias) | Selective reporting (reporting bias) | Other bias |
| --- | --- | --- | --- | --- | --- | --- | --- |
| Godfrey et al. (1985) | ? | ? | ? | ? | ? | + | + |
| Godfrey and Knight (1985) | ? | ? | ? | ? | ? | ? | ? |
| Yohman et al. (1988) | + | ? | ? | ? | - | ? | - |
| Wetzig and Hardin (1990) | + | ? | ? | ? | - | - | - |
| Steingass et al. (1994) | ? | ? | ? | ? | ? | ? | ? |
| Fals-Stewart and Lucente (1994) | ? | ? | ? | ? | ? | ? | - |
| Peterson et al. (2002) | ? | + | + | + | ? | - | - |
| Goldstein et al. (2005) | ? | ? | ? | - | - | - | - |
| Fals-Stewart and Lam (2010) | ? | ? | - | - | - | - | - |
| Rupp et al. (2012) | - | ? | ? | ? | - | - | - |
| Gamito et al. (2013) | ? | ? | ? | ? | - | ? | - |
| Gamito et al. (2014) | - | ? | + | ? | - | - | - |
| Eack et al. (2015) | ? | ? | ? | ? | - | - | - |
| Rass et al. (2015) | ? | + | - | - | - | - | - |
| Bell et al. (2016) | - | - | ? | ? | - | - | - |
| Brooks et al. (2016) | + | + | - | - | - | ? | - |
| Gamito et al. (2016) | - | + | + | - | - | - | - |
| Bell et al. (2017) | - | - | ? | - | ? | - | - |
| Brooks et al.(2017) | ? | ? | ? | - | - | ? | - |
| Gamito et al. (2017) | ? | + | + | ? | ? | ? | - |
| Gunn et al. (2018) | ? | ? | ? | ? | ? | ? | - |
| Hendershot et al. (2018) | - | - | - | - | - | - | - |
| Zhu et al. (2018) | ? | - | ? | ? | - | - | - |
| Khemiri et al. (2019) | - | - | - | - | - | - | - |
| Rezapour et al. (2019) | - | - | ? | ? | - | - | - |

*Note*. - indicates low risk of bias; + indicates high risk of bias; ? indicates unclear risk of bias.

**S2.** *Risk-of-bias Assessment of the Non-Randomized Study with the ROBINS-I Tool*

| Author (Year) | Baseline  confounding | Selection of  participants | Classification of  intervention | Deviation from  intended intervention | Missing  data | Measurement  of outcomes | Selection of  reported results |
| --- | --- | --- | --- | --- | --- | --- | --- |
| Hannon et al. (1989) | +/- | +/- | +/- | - | ? | - | +/- |

*Note*. - indicates low risk of bias; +/- indicates moderate risk of bias; + indicates serious risk of bias; ++ indicates critical risk of bias; ? indicates no information.

**S3.** *Full text articles excluded with reasons*

| **Full text articles excluded** | **Reasons** |
| --- | --- |
| Bates et al. (2004) | Does not include cognitive training |
| Bell et al. (2016) | Wrong study design |
| Berry et al. (2019) | Study Protocol – Full study not available |
| Frias-Torres et al. (2018) | Wrong study design |
| Goldman and Goldman (1988) | Wrong study design |
| Gordon et al. (1988) | Wrong study design |
| Grohman and Fals-Stewart (2003) | Unable to find |
| Grohman et al. (2002) | Unable to find |
| Kaag et al. (2017) | Without objective measures |
| Kalapatapu et al. (2013) | Does not include cognitive training |
| Kiluk et al. (2017) | No control group |
| Man (2018) | Age of participants |
| Manning et al. (2019) | Outcomes of interest |
| McGurk et al. (2010) | Wrong patient population |
| Moriyama et al. (2006) | Wrong study design |
| Passetti et al. (2011) | Wrong study design |
| Pedrero-Perez et al. (2013) | Wrong study design |
| Rezapour et al. (2015) | Wrong study design |
| Schulte et al. (2019) | No control group |
| Schulte et al. (2018) | No control group |
| Strickland et al. (2019) | Without objective measures |
| Sweeney et al. (2016) | No control group |
| Thompson and Filley (1989) | Wrong patient population |
| Wanmaker et al. (2018) | Age of participants |

# References

Bates, M. E., Barry, D., Labouvie, E. W., Fals-Stewart, W., Voelbel, G. & Buckman, J. F. (2004). Risk factors and neuropsychological recovery in clients with alcohol use disorders who were exposed to different treatments. Journal of Consulting and Clinical Psychology, 72(6), 1073–1080. <https://doi.org/10.1037/0022-006X.72.6.1073>

Bell, M. D., Vissicchio, N. A. & Weinstein, A. J. (2016). Visual and verbal learning deficits in Veterans with alcohol and substance use disorders. Drug and Alcohol Dependence, 159, 61–65. <https://doi.org/10.1016/j.drugalcdep.2015.11.007>

Berry, J., Jacomb, I., Lunn, J., Sedwell, A., Shakeshaft, A., Kelly, P. J., Sarrami, P., James, M., Russell, S., Nardo, T., Barker, D. & Holmes, J. (2019). A stepped wedge cluster randomised trial of a cognitive remediation intervention in alcohol and other drug (AOD) residential treatment services. BMC Psychiatry, 19. <https://doi.org/10.1186/s12888-019-2044-4>

Frias-Torres, C., Moreno-Espana, J., Ortega, L., Barrio, P., Gual, A. & Teixidor, L. (2018). Remediation therapy in patients with alcohol use disorders and neurocognitive disorders: A pilot study. Adicciones, 30(2), 93–99. <https://doi.org/http://dx.doi.org/10.20882/adicciones.757>

Goldman, R. S. & Goldman, M. S. (1988). Experience-dependent cognitive recovery in alcoholics: A task component strategy. Journal of Studies on Alcohol, 49(2), 142–148. <https://doi.org/http://dx.doi.org/10.15288/jsa.1988.49.142>

Gordon, S. M., Kennedy, B. P. & McPeake, J. D. (1988). Neuropsychologically impaired alcoholics: Assessment, treatment considerations, and rehabilitation. Journal of Substance Abuse Treatment, 5(2), 99–104. <https://doi.org/http://dx.doi.org/10.1016/0740-5472%2888%2990019-0>

Grohman, K. & Fals-Stewart. (2003). Computer-Assisted Cognitive Rehabilitation with Substance-Abusing Patients: Effects on Treatment Response. Journal of Cognitive Rehabilitation, 21(4), 10–17. <http://ovidsp.ovid.com/ovidweb.cgi?T=JS&PAGE=reference&D=psyc4&NEWS=N&AN=2004-11669-003>

Grohman, K., Fals-Stewart, W. & Bates, M. (2002). Cognitive rehabilitation for neuropsychologically impaired substance-abusing patients Posttreatment outcomes. Drug and Alcohol Dependence, 66(Suppl. 1), S70.

Kaag, A. M., Goudriaan, A., De Vries, T. J., Pattij, T. & Wiers, R. W. (2017). A high working memory load prior to memory retrieval reduces craving in non-treatment seeking problem drinkers. Psychopharmacology, 235(3), 695–708.

Kalapatapu, R. K., Lewis, D. F., Vinogradov, S., Batki, S. L. & Winhusen, T. (2013). Relationship of age to impulsivity and decision making: A baseline secondary analysis of a behavioral treatment study in stimulant use disorders. Journal of Addictive Diseases, 32(2), 206–216. <https://doi.org/10.1080/10550887.2013.795471>

Kiluk, B. D., Buck, M. B., Devore, K. A., Babuscio, T. A., Nich, C. & Carroll, K. M. (2017). Performance-based contingency management in cognitive remediation training: A pilot study. Journal of Substance Abuse Treatment, 72, 80–88. <https://doi.org/10.1016/j.jsat.2016.08.003>

Man, D. W. K. (2018). Virtual reality-based cognitive training for drug abusers: A randomised controlled trial. Neuropsychological Rehabilitation, 8, 1–18. <https://doi.org/10.1080/09602011.2018.1468271>

Manning, V., Garfield, J. B. B., Mroz, K., Campbell, S. C., Piercy, H., Staiger, P. K., Lum, J. A. G., Lubman, D. I. & Verdejo-garcia, A. (2019). Feasibility and acceptability of approach bias modification during methamphetamine withdrawal and related methamphetamine use outcomes. Journal of Substance Abuse Treatment, 106, 12–18. <https://doi.org/10.1016/j.jsat.2019.07.008>

McGurk, S. R., Schiano, D., Mueser, K. T. & Wolfe, R. (2010). Implementation of the thinking skills for work program in a psychosocial clubhouse. Psychiatric Rehabilitation Journal, 33(3), 190–199.

Moriyama, Y., Muramatsu, T., Kato, M., Mimura, M. & Kashima, H. (2006). Family history of alcoholism and cognitive recovery in subacute withdrawal. Psychiatry and Clinical Neurosciences, 60(1), 85–89. <https://doi.org/http://dx.doi.org/10.1111/j.1440-1819.2006.01464.x>

Passetti, F., Clark, L., Davis, P., Mehta, M. A., White, S., Checinski, K., King, M. & Abou-Saleh, M. (2011). Risky decision-making predicts short-term outcome of community but not residential treatment for opiate addiction. Implications for case management. Drug and Alcohol Dependence, 118(1), 12–18. <https://doi.org/10.1016/j.drugalcdep.2011.02.015>

Pedrero-Perez, E. J., de Leon, J. M., Lozoya-Delgado, P., Rojo-Mota, G., Llanero-Luque, M. & Puerta-Garcia, C. (2013). Prefrontal symptoms and personality disorders in substance abusers. Revista de Neurologia, 56(4), 205–213. <https://doi.org/10.33588/rn.5604.2012583>

Rezapour, T., Hatami, J., Farhoudian, A., Sofuoglu, M., Noroozi, A., Daneshmand, R., Samiei, A. & Ekhtiari, H. (2015). NEuro COgnitive REhabilitation for Disease of Addiction (NECOREDA) Program: From Development to Trial. Basic and Clinical Neuroscience, 6(4), 291–298.

Schulte, M. H. J., Kaag, A. M., Boendermaker, W. J., Brink, W. van den, Goudriaan, A. E. & Wiers, R. W. (2019). The effect of N-acetylcysteine and working memory training on neural mechanisms of working memory and cue reactivity in regular cocaine users. Psychiatry Research. Neuroimaging, 287, 56–59. <https://doi.org/10.1016/j.pscychresns.2019.03.011>

Schulte, M. H. J., Wiers, R. W., Boendermaker, W. J., Goudriaan, A. E., van den Brink, W., van Deursen, D. S., Friese, M., Brede, E. & Waters, A. J. (2018). Reprint of the effect of N-acetylcysteine and working memory training on cocaine use, craving and inhibition in regular cocaine users: Correspondence of lab assessments and Ecological Momentary Assessment. Addictive Behaviors, 83, 79–86. <https://doi.org/http://dx.doi.org/10.1016/j.addbeh.2018.03.023>

Strickland, J. C., Hill, J. C., Stoops, W. W. & Rush, C. R. (2019). Feasibility, acceptability, and initial efficacy of delivering alcohol use cognitive interventions via crowdsourcing. A lcoholism-Clinical and Experimental Research, 43(5), 888–899. <https://doi.org/10.1111/acer.13987>

Sweeney, M. M., Rass, O., Johnson, P. S., Strain, E. C., Berry, M. S., Vo, H. T., Fishman, M. J., Munro, C. A., Rebok, G. W., Mintzer, M. Z. & Johnson, M. W. (2016). Initial feasibility and validity of a prospective memory training program in a substance use treatment population. Experimental and Clinical Psychopharmacology, 24(5), 390–399. <https://doi.org/10.1037/pha0000091>

Thompson, L. L. & Filley, C. M. (1989). A pilot study of neuropsychological rehabilitation. Journal of Neurologic Rehabilitation, 3(3), 117–127. <https://doi.org/http://dx.doi.org/10.1177/136140968900300302>

Wanmaker, S., Leijdesdorff, S. M. J., Geraerts, E., van de Wetering, B. J. M., Renkema, P. J. & Franken, I. H. A. (2018). The efficacy of a working memory training in substance use patients: A randomized double-blind placebo-controlled clinical trial. Journal of Clinical and Experimental Neuropsychology, 40(5), 473–486. <https://doi.org/10.1080/13803395.2017.1372367>
